# Towards Generation of Cat States in Trapped Ions Set-Ups via FAQUAD Protocols and Dynamical Decoupling

**DOI:** 10.3390/e21121207

**Published:** 2019-12-09

**Authors:** Mikel Palmero, Miguel Ángel Simón, Dario Poletti

**Affiliations:** 1 Science and Math Cluster, Singapore University of Technology and Design, 8 Somapah Road, Singapore 487372, Singapore; 2 Departamento de Química-Física, Universidad del País Vasco UPV-EHU, B. Sarriena s/n, 48940 Leioa, Spain; miguelangel.simon@ehu.eus; 3 Engineering Product Development, Singapore University of Technology and Design, 8 Somapah Road, Singapore 487372, Singapore

**Keywords:** shortcuts to adiabaticity, quantum simulation, superposition states, dissipative many-body systems

## Abstract

The high fidelity generation of strongly entangled states of many particles, such as cat states, is a particularly demanding challenge. One approach is to drive the system, within a certain final time, as adiabatically as possible, in order to avoid the generation of unwanted excitations. However, excitations can also be generated by the presence of dissipative effects such as dephasing. Here we compare the effectiveness of Local Adiabatic and the FAst QUasi ADiabatic protocols in achieving a high fidelity for a target superposition state both with and without dephasing. In particular, we consider trapped ions set-ups in which each spin interacts with all the others with the uniform coupling strength or with a power-law coupling. In order to mitigate the effects of dephasing, we complement the adiabatic protocols with dynamical decoupling and we test its effectiveness. The protocols we study could be readily implemented with state-of-the-art techniques.

## 1. Introduction

The possibility of generating many-body entangled states has important consequences in metrology [1,2,3,4] and in quantum computation [5]. One approach to producing such states is to first prepare the system in a ground state easy to obtain with high fidelity, for example, in the presence of a strong magnetic field and then adiabatically transfer the state to the target ground state of a modified Hamiltonian, for example, by ramping down the magnetic field. However, in such approach, one encounters two main difficulties: the first is the presence of small avoided crossings, what makes it difficult to follow the ground state adiabatically without producing excitations in a finite time; the second is the presence of sources of dissipation which may also excite the system. To counter the first point, one could choose to evolve the system very slowly. However, for practical applications, it would be ideal to be able to prepare target states in times as short as possible. Moreover, it is clear that the longer the preparation of a state takes, the longer the dissipation will affect the system, thus driving it away from the target state. It is therefore necessary to use a strategy that allows, simultaneously, both the preparation of a target state quickly, reducing the possible excitations from the Hamiltonian driving, and the protection of the system from the effects of dissipation.

In our work we consider a system of spins coupled to each other via phonon-mediated interactions, a very tunable model for a quantum simulator. In fact, such a model can be realized both in cavity QED systems [6,7] and with trapped ions [8,9,10,11]. The fact that the interactions are mediated by the phonons significantly increases the tunability of the set-ups, but it also introduces a source of dissipation, which is the dephasing due to the phonons themselves. The preparation of target cat states in a trapped ion set-up was recently studied in Reference [12]. In this work they studied, both theoretically and experimentally, the evolution of ions in a Penning trap. They prepared a system in a product state in the presence of a large magnetic field, and then reduced the magnetic field to drive it to a cat state. In particular, they showed that tuning the magnetic field following a so-called local adiabatic (LA) approach [13] of the shortcuts to adiabaticity [14,15].

Here we aim to continue this line of research by combining two techniques: the use of a different method to design the time evolution of the magnetic field, namely the fast quasi adiabatic (FAQUAD) protocol [16], and the use of dynamical decoupling [17,18] to tame the effects of dephasing. We will also consider both the scenarios of trapped ions in a two-dimensional Penning trap [19] which results in equal interaction between all spins, and that of a linear Paul trap in which the interaction as a function of distance follows a power-law decay [9,11]. There are several arguments to justify the choice of the FAQUAD method over all the available methods in shortcut to adiabaticity techniques. Firstly, FAQUAD starts with a predetermined Hamiltonian and, by applying it, we only design the time dependence of our control parameter, which is known to be possible to produce in the lab. Other universal methods, in the sense that they can always produce a solution to speed up the adiabatic dynamics—such as the counter-diabatic [20] or the fast-forward [21] methods—rely on producing an additional field that will compensate for the excitations the original Hamiltonian produces. The type of field these methods will require are in principle not controllable and in general will not be possible to produce in a given experimental setup. Other methods that, similar to FAQUAD, start with a given Hamiltonian and solve the dynamics by designing the time evolution of the control parameter so that it satisfies the conditions for no excitations in boundary times include the LA method already tried in Reference [12], the invariant based inverse engineering [22], or the optimal control theory [23,24]. Invariant based techniques require analytical solvability since commutation relations have to be obtained between the Hamiltonian and the invariant. For the complex many-body systems studied here, it is impossible in practice to obtain an invariant to work with. A way out is working within the mean field approximation as in Reference [22]. However, this does not solve the dynamics for exact systems we are interested in, so we think this gives both FAQUAD and LA a strong edge. Finally, optimal control theory minimizes the final excitation by using brute force computation. The advantage of the FAQUAD method with respect to optimal control is that, although in this case it needs to be numerically calculated, it relies on solving a simple integral, so the protocols can be easily recalculated when needed for different parameters than the ones we use here as an example. In short, although the topic of created entangled states has attracted much attention, both from experimentalists [1,9,10,11,12,13] and from theorist trying to design shortcuts to improve the fidelities [12,13,22,23,25,26], the fidelities obtained so far have been moderate and the designed shortcuts have not been implemented other than the LA method in Reference [12]. Our aim is to produce a shortcut that will allow for better fidelities, also in the presence of dissipation, and yet at the same time, is relatively straightforward to implement in the lab. We stress here that the dissipation does not drive the system to a desired state (e.g., a thermal state or an entangled target state), which we aim to obtain as quickly as possible [27,28]. Instead, the dissipation has detrimental effects on the target state, because of which we need to find protocols which are as fast as possible and which could possibly mitigate the effect of the dissipation.

The manuscript is structured in the following manner: In Section 2 we describe the methods of shortcuts to adiabaticity [14] to design the optimized adiabatic protocols that we will use. In Section 3 we focus on a spin system with uniform all-to-all interactions, and we study the effectiveness of LA and FAQUAD protocols both for unitary and dissipative evolution. For the latter, we will also consider the effect of dynamical decoupling. In Section 4 we focus on a spin system with power-law interactions, and in Section 5 we draw our conclusions.

## 2. Local Adiabatic and FAQUAD Protocols

As mentioned in the introduction, it is possible to reduce the amount of excitation in the prepared state by designing an appropriate protocol for the time-dependence of the Hamiltonian control parameters. Here we give an introduction to the Local Adiabatic (LA) [13] and the FAst QUasi-ADiabatic (FAQUAD) [16] protocols. The main idea is to start from the adiabaticity condition which imposes that the change in a state should be much smaller when the energy gap between this states and another relevant state. This translates to

(1) $$\hbar \left|\frac{\langle \psi_{a}\left(t\right)|\partial_{t}\psi_{b}\left(t\right)\rangle }{E_{a}\left(t\right)-E_{b}\left(t\right)}\right|\ll 1,$$

where the $|\psi_{i}\rangle$ are two eigenstates and the $E_{i}$ the corresponding eigenenergies. The relevant states to consider are, for our application, the ground state and the first excited state which is coupled by the changing Hamiltonian (which, due to symmetries, could for instance be the second excited state of the instantaneous Hamiltonian H(t)). It is thus possible to distribute homogeneously in time the probability of transition between the two energy levels involved in the equation by imposing the following condition

(2) $$\hbar \left|\frac{\left|\psi_{a}\left(t\right)\right|\partial_{t}\psi_{b}\left(t\right)\rangle }{E_{a}\left(t\right)-E_{b}\left(t\right)}\right|=\hbar \left|\frac{\langle \psi_{a}\left(t\right)\left|\frac{\partial H}{\partial t}\right|\psi_{b}\left(t\right)\rangle }{{\left[E_{a}\left(t\right)-E_{b}\left(t\right)\right]}^{2}}\right|=c.$$

Then, rewriting the time vector as a function of the control parameter, $t=t(B_{\mu })$ (we label the control parameter as $B_{\mu }$ because in the following the control parameter will be the magnetic field $B_{\mu }$, by μ we indicate a particular time-dependence/protocol to vary it), Equation (2) gives the FAQUAD protocol

(3) $${\dot{B}}_{F}=\mp \frac{c}{\hbar }\left|\frac{{\left[E_{a}\left(B_{F}\right)-E_{b}\left(B_{F}\right)\right]}^{2}}{\langle \psi_{a}\left(B_{F}\right)\left|\frac{\partial H}{\partial B_{F}}\right|\psi_{b}\left(B_{F}\right)\rangle }\right|.$$

By simply integrating this equation, one could obtain the control parameter $B_{F}$ as a function of time [16]. Given the size of the systems we will study, the protocols will be evaluated numerically. The LA method, see Reference [13], stems from Equation (3), with an additional assumption that simplifies the calculation of the protocol. The assumption is that $\langle \psi_{a}\left(B\right)\left|\frac{\partial H}{\partial B}\right|\psi_{b}\left(B\right)\rangle =1$ for all times, which results in the equation for the parameter $B_{L}\left(t\right)$

(4) $${\dot{B}}_{L}=\mp \frac{c}{\hbar }\left|{\left[E_{a}\left(B_{L}\right)-E_{b}\left(B_{L}\right)\right]}^{2}\right|.$$

Since FAQUAD uses the adiabaticity condition more accurately, the ensuing protocol distributes the loss of adiabaticity better along the time evolution. In the following we will also consider a modification of the FAQUAD protocol. In Equation (3) we only considered the ground state and the first relevant excited state. However, the time-dependent Hamiltonian could couple, in a non-negligible manner, the instantaneous ground state to a few excited levels. We thus obtain

(5) $${\dot{B}}_{K}=\frac{c}{\hbar }{\left(\sum_{k=1}^{K}\left|\frac{\langle \psi_{g}\left(B_{K}\right)\left|\frac{\partial H}{\partial B_{K}}\right|\psi_{k}\left(B_{K}\right)\rangle }{{\left[E_{g}\left(B_{K}\right)-E_{k}\left(B_{K}\right)\right]}^{2}}\right|\right)}^{-1},$$

where the subscript *g* indicates the ground state, while the *k* enumerates the lowest excited states coupled to the instantaneous ground state. In this paper we considered up to K=5 relevant transitions to design the protocols and we refer to the FAQUAD protocols that consider *K* relevant transitions as FAQUAD-K.

## 3. Uniform All-To-All Interactions

We consider *N* ions in a trap with spins degrees of freedom coupled to a normal mode of the system via a spin-dependent optical dipole force. In particular, we focus on the case in which the optical dipole force is tuned such that the center of mass mode is uniformly coupled to all the ions/spins. This setup, with the addition of an external magnetic field $B_{\mu }\left(t\right)$ can be described by the Dicke Hamiltonian [29,30,31]

(6) $${\hat{H}}_{\;\mathrm{Dicke}\;}=-\frac{\hbar g_{0}}{\sqrt{N}}\left(\hat{a}+{\hat{a}}^{†}\right){\hat{S}}_{z}+B_{\mu }\left(t\right){\hat{S}}_{x}-\hbar \delta {\hat{a}}^{†}\hat{a},$$

where $a,a^{†}$ are the bosonic annihilation and creation operators, $B_{\mu }\left(t\right)$ is the time-dependent transverse magnetic field from the protocol μ=F,L,K (see Equations (3)–(5)), $g_{0}$ is the coupling between the spins and the center of mass mode and δ is the detuning between the optical dipole force and the center of mass mode. In Equation (6) we have used the collective operator ${\hat{S}}_{\alpha }=\left(1/2\right)\sum_{j}{\hat{\sigma }}_{j}^{\alpha }$, being *j* the label for each spin and ${\hat{\sigma }}_{j}^{\alpha }$ the Pauli matrices for α=x,y,z.

At this point it is possible to rewrite the Hamiltonian (6) as

(7) $$\hat{H}\left(t\right)=-\hbar \delta {\hat{b}}^{†}\hat{b}+\frac{J}{N}{\hat{S}}_{z}^{2}+B_{\mu }\left(t\right){\hat{S}}_{x},$$

where $\hat{b}=\hat{a}-\left[g_{0}/\left(\sqrt{N}\delta \right)\right]{\hat{S}}_{z}$ and $J=\hbar g_{0}^{2}/\delta$. Here the term $∼{\hat{b}}^{†}\hat{b}$ describes the phonons in a displaced potential, with a spin dependent displacement. In the limit of large detuning, $\left|\delta \right|\gg g_{0}/\sqrt{N}$, the bosonic mode can be adiabatically eliminated, leaving a purely spin system

(8) $${\hat{H}}_{\mathrm{LM}}\left(t\right)=\frac{J}{N}{\hat{S}}_{z}^{2}+B_{\mu }\left(t\right){\hat{S}}_{x},$$

which, for δ<0 is known as the ferromagnetic Lipkin model Hamiltonian. The Lipkin and the Dicke Hamiltonians show a similar behavior—for large magnetic field, the ground state is such that the spins are polarized in the *x* direction, while for small magnetic field they are in a symmetric superposition of all spins pointing up plus all spins pointing down in the *z* direction. However, in the latter case, the energy gap is small and it is thus difficult to prepare such state via adiabatic driving.

Using the Lipkin model to describe the physical setup has two important advantages that significantly simplify the study. First it eliminates the bosonic degree of freedom and, second, it allows us to further reduce the relevant Hilbert space as the spin states will belong to the Dicke manifold composed of N+1 states only, each a symmetric of superposition of the spin states with the same magnetization.

More precisely, for *N* spins and $\bar{n}$ maximum bosonic occupation, the size of the relevant Hilbert space for the Dicke Hamiltonian in Equation (6) is $D_{D}=2^{N}\left(\bar{n}+1\right)$, while for the Lipkin model Equation (8) it is $D_{\mathrm{LM}}=2^{N}$. However, if the initial condition is in the symmetric sector in which all spins are prepared in the same state, we notice that the state will only evolve within this symmetry sector. Hence the dimensionality of the relevant Hilbert spaces can be reduced to $D_{D}^{s}=\left(N+1\right)\left(\bar{n}+1\right)$ and $D_{\mathrm{LM}}^{s}=N+1$.

The experimental realization of such a setup, however, comes with dissipative effects. Following Reference [12], we will consider that the open dynamics is mostly subject to dephasing, which can be described by a master equation of the Gorini-Kossakowski-Sudarshan-Lindblad form [32,33]

(9) $$\frac{d\hat{\rho }}{dt}=-\frac{i}{\hbar }\left[\hat{H},\hat{\rho }\right]+\frac{\Gamma }{2}\sum_{i=1}^{N}\left({\hat{\sigma }}_{i}^{z}\hat{\rho }{\hat{\sigma }}_{i}^{z}-\hat{\rho }\right),$$

such that the dephasing is produced by single site $\sigma^{z}$ operators.

### 3.1. Closed System Scenario

**Figure 1:**
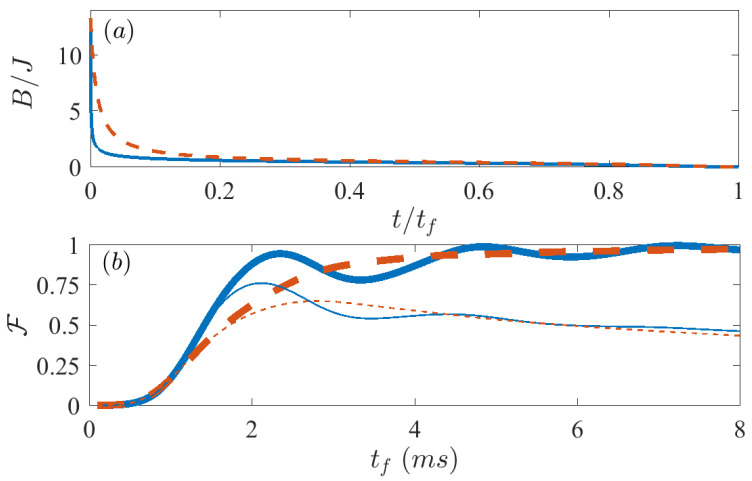
(**a**) The protocols derived from Equations (3) and (4) and (**b**) the fidelity F after evolving the master equation (9) using the Lipkin Hamiltonian (8). Solid blue lines are for FAQUAD and dashed red lines for local adiabatic (LA). Thick lines show the fidelities for the unitary evolution, that is, Γ=0 s${}^{-1}$, whereas the thin lines are for the open system with dephasing Γ=120 s${}^{-1}$. The rest of the parameters are, N=6, $B_{0}/\left(2\pi \right)=7$ KHz, J=0.55N KHz and δ/(2π)=−4 KHz.

We first consider an ideal case in which there is no dissipation, that is, Γ=0. We take the protocols designed using the FAQUAD and LA methods in Equations (3)–(5), and calculate the corresponding time evolution solving the Schrödinger equation using a Runge-Kutta solver. We start from the paramagnetic ground state for a large initial magnetic field ($B_{0}/\left(2\pi \right)=7$ KHz), and aim to reach the ferromagnetic superposition state at final time when $B_{\mu }=0$. As a representative case, we run a simulation for N=6 spins in the Lipkin model (8), with parameters that can be reproduced in state-of-the-art labs [12]. The dependence on time of the magnetic field for the FAQUAD and LA protocols are depicted in Figure 1a respectively by the blue solid line and the red dashed line. The thick lines in Figure 1b depict the fidelities obtained after the evolution following the LA (dashed red line) and FAQUAD (solid blue line) approaches as a function of final time. More precisely, we evolve the initial state with a protocol determined by the chosen final time and for each of these final times we measure the final fidelity. We define the fidelity as $F=(\rho |\psi_{Target}\rangle \langle \psi_{Target}|)$ where ρ is the actual state reached at final time $t_{f}$ and $\psi_{Target}$ is the superposition ground state of the Lipkin Hamiltonian at $B_{\mu }=0$ [34].

Unlike for the LA protocol, in which case the fidelity increases monotonously with the final time, for FAQUAD-based protocols the fidelity shows an oscillatory behavior. An explanation to this oscillatory behavior is given in Reference [16]. When the evolution is nearly adiabatic, the wavefunction can be approximated by the adiabatic perturbation theory. For the FAQUAD dynamics and approximating to only 2-level systems, the population transfer between levels can be approximated to a simple oscillatory function with a frequency proportional to the energy gap between both levels. If more levels were involved during the evolution, the different frequencies will in general be incommensurate, so the oscillatory behavior would difficult to see or at least would show a more irregular behavior. For this case the excitations are clearly concentrated in a single excited state (when we try FAQUAD protocols that consider more relevant transitions the results do not improve), therefore we can clearly see this smooth oscillations. This implies that while FAQUAD allows us to reach high fidelities at shorter times, it is also possible to find final times for which LA performs better. However, since the general objective is to obtain as good fidelities as possible in the shortest possible final time, we can state that FAQUAD already brings a clear improvement with respect to LA. For instance, if we fix a target fidelity of F=0.99, we see that using FAQUAD protocol it is possible to reach such value at final times $t_{f}∼4.8$ ms, while using LA protocol only reaches that same fidelity only at $t_{f}=12.4$ ms. It is thus possible to reach the same level of fidelity in a time 2.6 times shorter. This result can be further improved, for instance, we have found that for the same number of spins, by varying the system parameters in the vicinity of the values used in Figure 1b FAQUAD can require a time which is even 4 times shorter compared to LA.

### 3.2. Performance of the Unitary Protocols in the Open System Scenario

In experimental set-ups, dephasing can affect the fidelity in a detrimental way. Since the FAQUAD protocol (Equation (3)) can reach higher fidelities in a shorter time, it could potentially perform better than the LA protocol, Equation (4). However, one protocol could drive the state for longer periods to states more easily affected by dissipation and thus result in worse performance. Here we analyze quantitatively the effect of dephasing on the resulting fidelity when using the FAQUAD or LA protocols described in Equations (3) and (4), which, in the previous subsection were designed for, and tested in, the unitary case.

Since the Lindblad dissipator acts independently on the local spins, unlike the unitary Dicke or Lipkin Hamiltonians (Equations (6) and (8)), which are purely functions of the collective spins, the evolution of the system cannot be solely described by the symmetric subspaces analyzed in Section 3.1. Due to this, in order to have an accurate description of the system evolution using the Dicke Hamiltonian (6), one would need a vector space of dimension $2^{2N}{\left(\bar{n}+1\right)}^{2}$ or of dimension $2^{2N}$ when using the Lipkin Hamiltonian (8). In the following we will concentrate on the latter.

We apply again the theory in Section 2 for the Lipkin model (8), and use it to solve the open dynamics described by the master Equation (9). While the protocols are computed only taking into account the unitary part of the master equation, if they are effective over a short evolution they should also be less affected by dissipation. In particular, since dephasing is a cumulative effect, we expect that at short times the protocols will perform similarly to the closed system scenario. For longer times instead, we expect a decay in the fidelity caused by dephasing. The thin lines in Figure 1b show precisely this, where the results with protocols from FAQUAD are depicted by a thin blue solid line, while from LA by the thin red dashed line. At short final times $t_{f}$, the evolution of fidelity versus $t_{f}$ is identical for the dynamics in a closed (thick lines) or open system scenario (thin lines), but at around 2 ms the respective curves diverge and the peak of fidelity for open systems occurs at much lower values of $t_{f}$. After this maximum, the fidelity for the open system case decays almost monotonously versus the final time, whereas the one for the closed system approaches unit fidelity. For the parameters simulated in Figure 1b, the maximum fidelity with the FAQUAD protocol is $F_{F}=0.7628$, whereas the maximum fidelity for the LA protocol is $F_{LA}=0.6514$, which implies that the use of the FAQUAD protocol results in a 11% increase in fidelity.

**Figure 2:**
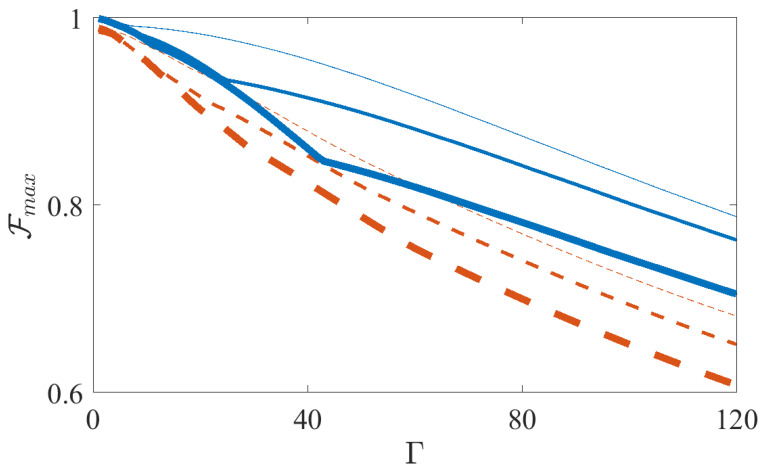
Maximum fidelity $F_{max}$ vs the value of the dephasing Γ after evolving the master Equation (9) using the Lipkin Hamiltonian (8). Solid blue lines are for FAQUAD and dashed red lines for LA, and the increasing thickness of the lines mean increasing system size, namely N=4, N=6 and N=8. The remaining parameters are, $B_{0}/\left(2\pi \right)=7$ KHz, J=0.55N KHz and δ/(2π)=−4 KHz.

In Figure 2 we show how the value of the maximum fidelity attainable $F_{max}$ depends on the magnitude of dephasing for different system sizes and on the protocol used, in particular FAQUAD (Equation (3)) or LA (Equation (4)). In Figure 2 we notice that for a given system size, protocols from FAQUAD (blue solid lines), perform better than from LA (red dashed lines). However, we also observe that as the system size increases (N=4,6 and 8, from thinner to thicker lines) the maximum fidelity decreases.

### 3.3. Dynamical Decoupling of the Dephasing

In References [17,18], it was shown that the use of additional terms to the Hamiltonian could result in filtering out unwanted effects of the system-bath interactions, what is known as “dynamical decoupling”. In this case, because in our effective Hamiltonian (8) we have a term proportional to $S_{z}^{2}$, we will add a dynamical decoupling term, proportional to $S_{y}^{2}$. For the Lipkin Hamiltonian with dephasing, and inspired by Reference [35], we notice that the addition of a term proportional to $S_{y}^{2}$ can be effective for dynamical decoupling.

To effectively obtain such $S_{y}^{2}$ term, we propose to introduce an independent spin-boson field using a new pair of lasers. The Dicke Hamiltonian with the new term ($\hat{c}$) will take the form

(10) $$\begin{array}{cc} {\hat{H}}_{\mathrm{Dicke}}^{\prime }/\hbar = & -\frac{g_{0}}{\sqrt{N}}\left(\hat{a}+{\hat{a}}^{†}\right){\hat{S}}_{z}+B_{\mu }\left(t\right){\hat{S}}_{x}-\delta {\hat{a}}^{†}\hat{a}\\ \; & -\frac{g_{0}}{\sqrt{N}}\left(\hat{c}+{\hat{c}}^{†}\right){\hat{S}}_{y}-\delta^{\prime }{\hat{c}}^{†}\hat{c}. \end{array}$$

If we now rewrite the bosonic mode as in Section 3, we get

(11) $$\begin{array}{cc} \hat{H}\left(t\right)= & -\delta {\hat{b}}^{†}\hat{b}+\frac{J}{N}{\hat{S}}_{z}^{2}+B\left(t\right){\hat{S}}_{x}\\ \; & -\delta {\hat{d}}^{†}\hat{d}+\frac{J^{\prime }}{N}{\hat{S}}_{y}^{2}, \end{array}$$

where, as in Section 3, $\hat{b}=\hat{a}-\left[g_{0}/\left(\sqrt{N}\delta \right)\right]{\hat{S}}_{z}$ and $J=\hbar g_{0}^{2}/\delta$ and for the additional introduced field $\hat{d}=\hat{c}-\left[g_{0}/\left(\sqrt{N}\delta^{\prime }\right)\right]{\hat{S}}_{y}$ and $J^{\prime }=\hbar g_{0}^{2}/\delta^{\prime }\equiv N\omega \sin\left(\frac{\pi t}{t_{f}}\right)$. We introduced the final equivalence so that the dynamical decoupling term will have an optimizing constant ω and we chose a sinusoidal time-dependence so that the dynamical decoupling term will be zero at initial and final times. The large detuning limit for the new field will require $\omega \sin\left(\frac{\pi t}{t_{f}}\right)\ll \frac{g_{0}}{\sqrt{N}}$, so for a small ω we can always rewrite the Hamiltonian in the Lipkin model form

(12) $${\hat{H}}_{\mathrm{LM}}^{\prime }\left(t\right)=\frac{J}{N}{\hat{S}}_{z}^{2}+\omega \sin\left(\frac{\pi t}{t_{f}}\right){\hat{S}}_{y}^{2}+B\left(t\right){\hat{S}}_{x}.$$

**Figure 3:**
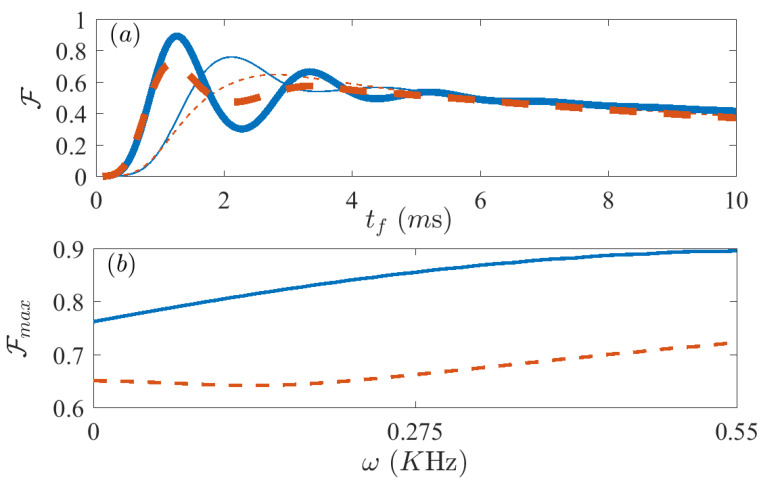
Fidelity F after evolving the master Equation (9) using the dynamically decoupled Lipkin Hamiltonian (12). (**a**) Displays the fidelity vs final time $t_{f}$ for the value of ω with best absolute fidelity (thick lines) and for the case without dynamical decoupling (thin lines), and (**b**) shows the maximum value of the fidelity at the first peak for each value of ω between 0 KHz and 0.55 KHz, Both panels compare results for the FAQUAD (solid blue) and LA (dashed red) protocols. Parameters are, N=6, $B_{0}/\left(2\pi \right)=7$ KHz, J=0.55N KHz and δ/(2π)=−4 KHz, Γ=120 s${}^{-1}$.

We use this Hamiltonian in Figure 3, mapping the optimizing parameter in the range ω∈[0,0.55] KHz. In Figure 3a we show the fidelity versus final time $t_{f}$, both without dynamical decoupling (ω=0 KHz, thin lines) and with dynamical decoupling (ω=0.55 KHz), both for FAQUAD (solid blue lines) and for LA (red dashed lines). In Figure 3b we plot the maximum value of the fidelity at the highest peak versus the optimizing parameter ω. Again the blue solid line reflects the results for FAQUAD, while the red dashed line those for LA. Here we observe that the maximum fidelity we can obtain for ω=0.55 KHz is $F_{\mathrm{FAQ}}=0.8968$ for the FAQUAD protocol, a 13.5% improvement and $F_{\mathrm{LA}}=0.7234$ for LA, corresponding to a 7% improvement. We note that we chose not to explore beyond ω=0.55 KHz because otherwise the Hamiltonian that we use may not satisfying the condition to adiabatically eliminate the bosonic mode (see discussion before Equation (12)).

**Figure 4:**
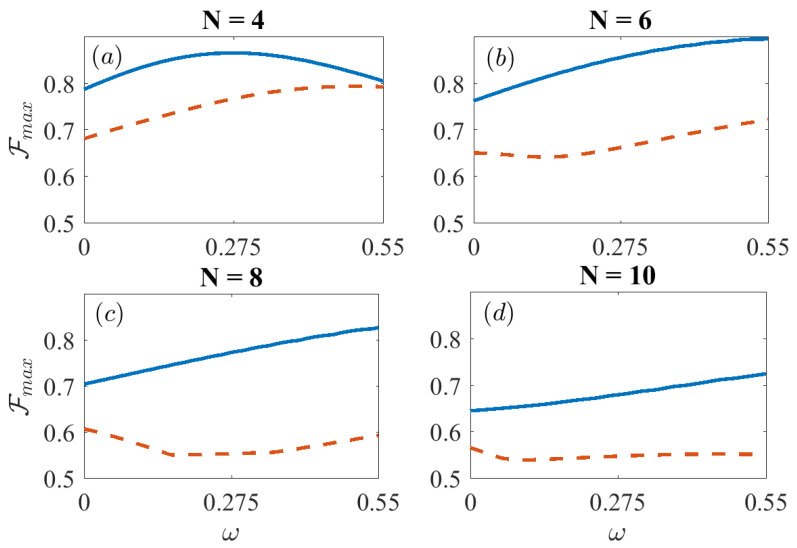
Maximum value of the fidelity after evolving the master Equation (9) using the dynamically decoupled Lipkin Hamiltonian (12) vs. parameter ω. We compare the fidelities for the FAQUAD (solid blue) and LA (dashed red) protocols for different chain sizes, ranging from N=4 to N=10. Parameters are, $B_{0}/\left(2\pi \right)=7$ KHz, J=0.55N KHz and δ/(2π)=−4 KHz, Γ=120 s${}^{-1}$.

In Figure 4, we study the effect of the chain size. Similarly to Figure 3 we plot the maximum fidelity $F_{max}$ versus the dynamical decoupling magnitude ω for chains of size ranging between N=4 and N=10. As the size grows, the maximum fidelity with no dynamical decoupling decreases with the system size. A possible explanation to this drop in fidelities for growing system sizes can be found in the orthogonality catastrophe arising from the quantum speed limit as recently studied by Fogarty et al. in Reference [36]. However, for the FAQUAD protocols studied we observe that the fidelity can (sometimes significantly) be increased thanks to dynamical decoupling.

## 4. Power-Law Interactions

Until now we have considered the scenario in which the interaction between the spins is uniform. In trapped ions setups the interaction can also be of power-law form with a tunable exponent. For instance, in Reference [10,11], the authors were able to realize the long range Ising Hamiltonian

(13) $${\hat{H}}_{\mathrm{Ising}}=\sum_{i<j}J_{i,j}{\hat{\sigma }}_{i}^{x}{\hat{\sigma }}_{j}^{x}+B_{\mu }\left(t\right)\sum_{i}{\hat{\sigma }}_{i}^{y},$$

where $J_{i,j}=\frac{J_{max}}{{\left|i-j\right|}^{\alpha }}$. As mentioned, the exponent of the long range interaction α can be tuned within a certain range. Here we will consider the two values α=1.2 and α=0, the latter for comparison purposes with the previous results using the Lipkin Hamiltonian (8). For a clearer comparison with previous results, we will choose similar values of the parameters, so that the transverse magnetic field $B_{\mu }$ will decay from an initial value $B_{\mu }\left(0\right)/\left(2\pi \right)=7$ KHz to a final value $B_{\mu }\left(t_{f}\right)=0$ KHz and we will also choose a similar interaction $J_{max}=-0.55$ KHz. Similar to the Spin-Boson model, in the presence of a dominant transverse field, the ground state is initialized with all spins aligned in the *y* direction. As the magnetic field is decreased to 0, the ground state becomes a degenerate ferromagnetic state (we consider *J* to be negative) in the *x* direction.

To account for the external noise, in this system we will consider a local dephasing ${\hat{\sigma }}_{i}^{x}$ which is in the same direction of the spin-spin interaction, with a master equation

(14) $$\frac{d\hat{\rho }}{dt}=-\frac{i}{\hbar }\left[{\hat{H}}_{\mathrm{Ising}},\hat{\rho }\right]+\frac{\Gamma }{2}\sum_{i=1}^{N}\left({\hat{\sigma }}_{i}^{x}\hat{\rho }{\hat{\sigma }}_{i}^{x}-\hat{\rho }\right).$$

We will study the performance of the LA and FAQUAD protocols designed for the unitary Hamiltonian (13). As we did before for the Lipkin model, we will also add a dynamically decoupling term. For this case we will add an oscillating term proportional ${\hat{\sigma }}_{i}^{z}{\hat{\sigma }}_{j}^{z}$ such that

(15) $$\begin{array}{cc} {\hat{H}}_{\mathrm{Ising}}^{\prime } & =\sum_{i<j}J_{i,j}{\hat{\sigma }}_{i}^{x}{\hat{\sigma }}_{j}^{x}+B_{\mu }\left(t\right)\sum_{i}{\hat{\sigma }}_{i}^{y}\\ \; & +\omega \sin\left(\frac{\pi t}{t_{f}}\right)\sum_{i<j}\frac{1}{{\left|i-j\right|}^{\tilde{\alpha }}}{\hat{\sigma }}_{i}^{z}{\hat{\sigma }}_{j}^{z}, \end{array}$$

where we consider in principle a different decay rate $\tilde{\alpha }$ as for the unitary Hamiltonian.

**Figure 5:**
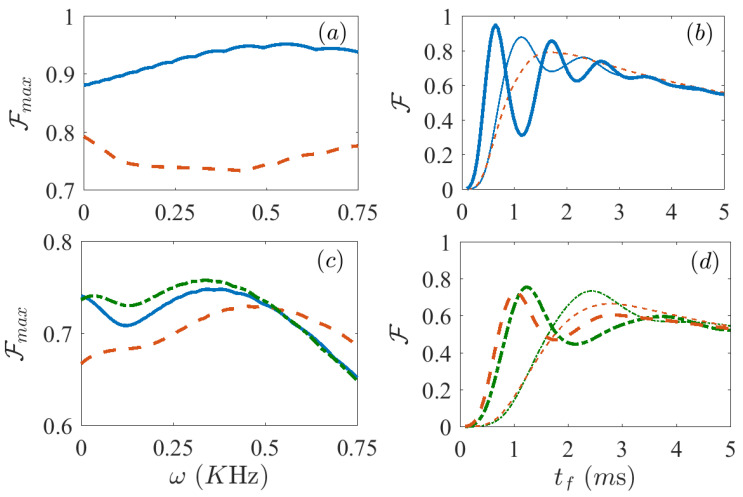
Fidelity F after evolving the master Equation (14) using the dynamically decoupled Ising Hamiltonian (15) for α=0 (upper panels) and α=1.2 (lower panels). (**a**,**c**) show the maximum value of the fidelity at the first peak for each value of ω between 0 KHz and 0.75 KHz, while (**b**,**d**) display the fidelity vs final time $t_{f}$ for the value of ω with best absolute fidelity (thick lines) and for the case without dynamical decoupling (thin lines). In panels (**a**–**c**) the solid blue lines are results for FAQUAD and dashed red lines for LA. In panel (**c**) we additionally have dash-dotted green lines for FAQUAD-4, the FAQUAD protocol that considers up to 4 relevant energy level transitions. In panel (**d**), solid blue lines are for FAQUAD4 and dashed red lines for LA. Parameters are, N=6, $B_{0}/\left(2\pi \right)=7$ KHz, $J_{max}=0.55$ KHz, $\tilde{\alpha }=0$ and Γ=120 s${}^{-1}$.

In Figure 5 we depict the fidelities after the evolutions using LA and FAQUAD protocols obtained for the unitary Hamiltonian (13), with and without a dynamically decoupling term. In panels (a,c) we plot the maximum fidelity versus the magnitude of dynamical decoupling ω∈[0,0.75] KHz for FAQUAD (blue solid line), LA (red dashed line) and FAQUAD-4 (yellow dot-dashed line). In panels (b,d) we show only the results corresponding to no dynamical decoupling (thin lines) and with ω corresponding to the highest fidelity (note that for panel (b) the maximum fidelity for LA is obtained already for ω=0). Panels (a,b) are for α=0, while panels (c,d) for α=1.2, that is, with a power-law potential. For α=0, the best fidelities obtained before applying dynamical decoupling with a dephasing Γ=120 s${}^{-1}$ are $F_{F}=0.8813$ and $F_{L}=0.7234$, about 16% better for the FAQUAD protocol. Once we introduce the dynamical decoupling term, the FAQUAD protocol can reach a maximum fidelity up to a 7% higher $F_{F}=0.9523$ when ω=553.7 Hz, whereas the result for LA does not improve for the parameters explored.

**Figure 6:**
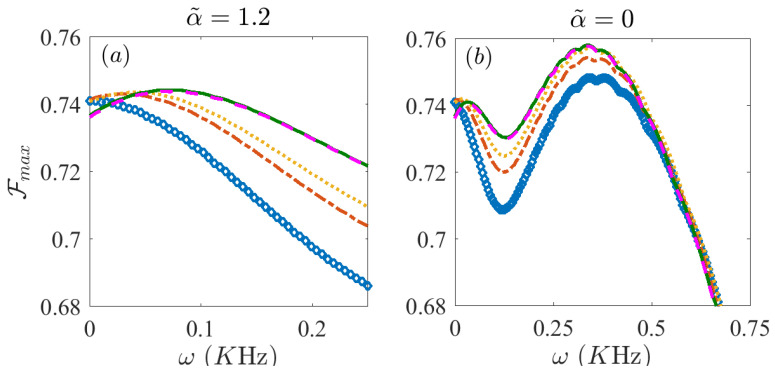
Maximum fidelity $F_{max}$ vs ω for the FAQUAD protocols considering a different number of meaningful transitions for $\tilde{\alpha }=1.2$ in panel (**a**) and $\tilde{\alpha }=0$ in panel (**b**). Blue diamonds are for FAQUAD-1, red dash-dotted line for FAQUAD-2, yellow dotted line for FAQUAD-3, solid green line for FAQUAD-4 and magenta dashed line for FAQUAD-5. Parameters are, N=6, $B_{0}/\left(2\pi \right)=7$ KHz, $J_{max}=0.55$ KHz and Γ=120 s${}^{-1}$.

Until this point, we have considered uniform all-to-all interactions and, for all these cases, the use of FAQUAD-K, see Equation (5), would not result in sizeable improvements in the fidelities. However, with a decaying long range interaction with α=1.2, we have tested for up to 5 relevant levels and the best fidelities are obtained for the FAQUAD protocol that considers up to 4 meaningful level transitions, that is, FAQUAD-4 (more details given in the discussion of Figure 6). Without dynamical decoupling, the highest fidelities are $F_{4}=0.7367$ and $F_{L}=0.6674$, almost a 7% better for the FAQUAD-4 protocol. Introducing dynamical decoupling, the best results are obtained for ω=337.25 Hz and ω=448 Hz respectively, reaching maximum fidelities of $F_{4}=0.7582$, 2% improvement and $F_{L}=0.7298$, 6% improvement.

The analysis of the dynamical decoupling for different types of protocols FAQUAD-K, for K=1 to 5 is done in Figure 6. In Figure 6a we show the comparison of the maximum fidelity of the different FAQUAD-K protocols versus the parameter ω when the interaction in the dynamically decoupling term $\tilde{\alpha }$ is the same as the interaction in the unitary Hamiltonian, that is, $\tilde{\alpha }=\alpha =1.2$. In Figure 6b we consider the case in which the spatial dependence of the interaction is different for the dynamical decoupling compared to the interaction term. In fact we compare the performance of the different FAQUAD-K protocols when $\tilde{\alpha }=0$, an analysis closely connected to Figure 5c. Interestingly, for both $\tilde{\alpha }=1.2$ and $\tilde{\alpha }=0$, FAQUAD-K protocols with larger K reach a smaller maximum fidelity ($F_{max}$) in the absence of dynamical decoupling (ω=0) but they perform much better with the dynamical decoupling term in Equation (15). By comparing panels (a) and (b) in Figure 6 we can also clearly observe that in this case a non decaying interaction for the dynamical decoupling performs, for this set-up and parameters, better than implementing the same space-dependence of the interaction as in the unitary Hamiltonian.

## 5. Conclusions

We have studied the effectiveness of different protocols in producing cat states with good fidelity. We have considered setups that can be realized experimentally with trapped ions, both with uniform and power-law interactions. We have shown that FAQUAD protocols perfom better than LA protocols in providing final states with high fidelity in shorter times. Moreover, we have shown how important this is when considering the effect of dephasing too. In fact, since FAQUAD protocols result in higher fidelities at short final times, the system has been under the influence of dephasing for a shorter period. This improvement is specially notable in the uniform interaction case. For instance, for the study case in the Lipkin model we observed an improvement of an 11% in fidelity. In the power law interaction case, we observed an improvement of 7% with respect to the LA protocol.

We were also able to further improve the fidelity of the target states by introducing an additional field perpendicular to the coupling to the bath to dynamically decouple the system from the environment. Notably, the presence of this additional term improves the fidelity for both FAQUAD and LA based protocols. In the cases studied here, we obtain an increase in fidelty up to 13% fidelity. Additionally, these larger maximum fidelities are reached at even shorter final times.

We have also considered models with spin interaction decaying as a power law, instead of a uniform all-to-all coupling. In this cases, we have observed that higher maximum fidelities can be reached with FAQUAD-K protocols, which is an extension of the FAQUAD protocol that takes into account the first *K* relevant excited states. We have observed that protocols with higher *K* can lead to an important improvement of the performance, especially in presence of dynamical decoupling. Interestingly, the dependence in space of the interaction for the dynamical decoupling term could be different from that of the spin-interactions in the Hamiltonian, and we have observed that a more uniform interaction in the dynamical decoupling term could help increase the fidelity.

In future works we could study larger system sizes. While the simulation of the dissipative dynamics is particularly demanding for a large number of spins, the computation of the protocol for the magnetic field only depends on the Hamiltonian (e.g., unitary evolution) and hence one can compute it for larger system sizes so that it could be tested in experiments. Although here we add the dynamical decoupling terms to improve the fidelity of the driving in the open system, the protocols for the ramp down of the magnetic field are not specifically designed to optimize the open dynamics. Some approaches have been tried to design shortcuts for open systems, for example using dynamical invariants [27,37] or counter diabatic driving [38,39]. However, these approaches require the ability to analytically solve the dynamics. Here, given the complexity of the many-body system, we would need a method that allows for a purely numerical design of the protocol. Another possible future direction could be developing such a shortcut technique, for example, reformulating the FAQUAD for open systems and implementing it for the cases discussed here.
